# Long non-coding RNA IGFBP7-AS1 accelerates the odontogenic differentiation of stem cells from human exfoliated deciduous teeth by regulating IGFBP7 expression

**DOI:** 10.1007/s13577-022-00763-9

**Published:** 2022-08-29

**Authors:** Dan Wang, Ningxin Zhu, Fei Xie, Man Qin, Yuanyuan Wang

**Affiliations:** Department of Pediatric Dentistry, School and Hospital of Stomatology, Peking University, #22 Zhongguancun South Avenue Nandajie, Haidian District, Beijing, 100081 China

**Keywords:** Odontogenic differentiation, SHED, IGFBP7-AS1, IGFBP7, Natural antisense transcripts (NATs)

## Abstract

Stem cells from human exfoliated deciduous teeth (SHED) are attractive seed cells for dental tissue engineering. We identified the effect of the long noncoding RNA insulin-like growth factor-binding protein 7 antisense RNA 1 (lncRNA IGFBP7-AS1) in vivo and its underlying mechanism during SHED odontogenic differentiation. IGFBP7-AS1 and insulin-like growth factor-binding protein 7 (*IGFBP7*) were overexpressed using lentiviruses. *IGFBP7* expression was knocked down with small interfering RNA. The effect of IGFBP7-AS1 in vivo was confirmed by animal experiments. The effect of *IGFBP7* on SHED odontogenic differentiation was assessed with alkaline phosphatase staining, alizarin red S staining, quantitative reverse transcription-PCR, and western blotting. The relationship between IGFBP7-AS1 and *IGFBP7* was confirmed by quantitative reverse transcription–PCR and western blotting. IGFBP7-AS1 promoted SHED odontogenesis in vivo, and regulated the expression of the coding gene *IGFBP7* positively. Inhibiting *IGFBP7* led to suppress SHED odontogenic differentiation while *IGFBP7* overexpression had the opposite effect. IGFBP7-AS1 enhanced the stability of *IGFBP7*. IGFBP7-AS1 promoted SHED odontogenic differentiation in vivo. The underlying mechanism may involve the enhancement of *IGFBP7* stability. This may provide novel potential targets for dental tissue engineering.

## Introduction

Stem cells from human exfoliated deciduous teeth (SHED) are attractive seed cells in tissue engineering because of their high proliferation ability and fewer ethical issues [1]. As one of the most commonly used mesenchymal stem cells, SHED have self-renewal ability and the capacity for multi-lineage differentiation to nerve tissue, bone tissue, and dental tissue when grown under defined culture conditions [2–4]. Realizing SHED differentiation into odontoblasts is the key point in the field of tooth regeneration. Long noncoding RNAs (lncRNAs), which are > 200-bp long, participate in various biological and pathological processes and regulate gene expression [5]. However, lncRNA regulation of odontogenesis in SHED is poorly defined. Via RNA sequencing experiments, we previously found that expression of the lncRNA insulin-like growth factor binding protein 7 antisense RNA 1 (IGFBP7-AS1) was gradually upregulated during SHED odontogenesis. Nevertheless, the effect of IGFBP7-AS1 on SHED odontogenesis and its mechanism is unknown.

Natural antisense transcripts (NAT) are endogenous transcripts complementary to other transcripts in organisms [6]. They can form double-stranded RNA by base complementary pairing with sense RNA [7]. Numerous protein-coding mRNAs have NATs, most of which appear to be ncRNAs [8]. An antisense lncRNA is defined as one that is > 200-bp long. Antisense lncRNAs regulate gene expression in the following four ways: transcriptional inhibition, RNA–DNA interaction (chromatin remodeling), nuclear RNA–RNA interaction, and cytoplasmic RNA–RNA interaction [9]. In this manner, antisense lncRNAs regulate sense transcript expression at the transcriptional and post-transcriptional levels. For example, lncRNAs enhanced the translation function of *Zeb2* and downregulated the expression of epithelial cadherin by blocking the *Zeb2* 5′ untranslated region (5′UTR) splice site [10]. The antisense lncRNA of ubiquitin carboxyterminal hydrolase L1 (Uchl1) activates protein synthesis by specifically complementing the 5′UTR end of *Uchl1* mRNA and affecting short interspersed nuclear elements [11]. IGFBP7-AS1 is located on chromosome 4 in humans, and is the antisense transcript of insulin-like growth factor binding protein 7 (IGFBP7). IGFBP7 is a secreted protein of the low-affinity IGFBP family, and most of its functions are independent of other IGF or IGF receptors. IGFBP7 is associated with the regions of bone and dentine deposition [12].

In the present study, we confirmed the odontogenic effect of IGFBP7-AS1 in vivo via animal experiments. Mechanistically, IGFBP7-AS1 regulated *IGFBP7* expression levels by enhancing *IGFBP7* stability. In summary, we identified the effect of IGFBP7-AS1 in vivo and its mechanism during SHED odontogenic differentiation.

## Materials and methods

### Cell culture and odontogenic differentiation

SHED were kindly provided by Oral Stem Cell Bank (Beijing Tason Biotech Co. Ltd., Beijing, China, http://www.kqgxb.com) and were from children aged 5–7 years. The SHED were cultured as previously described [13]. Our experiments were approved by the Ethics Committee of the Peking University School and Hospital of Stomatology, Beijing, China (approval number: PKUSSIRB-201732003). Stage P3 − P6 SHED were used for the experiments. To induce odontogenic differentiation, 70–80% confluent SHED were exposed to osteogenic media (OM) comprised of 0.01 mM dexamethasone disodium phosphate, 0.1 mM L-ascorbic acid phosphate, and 1.8 mM monobasic potassium phosphate (Sigma-Aldrich, MO, USA). The OM was changed every 2 days.

### Lentivirus infection

EF-1aF/GFP and Puro lentiviruses were constructed by GenePharma (Shanghai, China) to induce IGFBP7-AS1 and *IGFBP7* overexpression separately. SHED were transfected with lentiviruses (multiplicity of infection [MOI]: 20) to upregulate IGFBP7-AS1 levels and lentiviruses (MOI: 50) to upregulate *IGFBP7* levels. The lentivirus medium contained polybrene (5 mg/mL) to improve the infection efficiency. In addition, the medium was changed after 8 h.

### RNA oligoribonucleotides and cell transfection

RNA oligoribonucleotides (e.g., small-interfering RNAs [siRNAs] targeting lncRNA IGFBP7 and siRNA control [siNC]) were purchased from GenePharma (Shanghai, China). The sequences of siRNA IGFBP7 were as follows: GGGUCACUAUGGAGUUCAATT (sense), UUGAACUCCAUAGUGACCCTT (antisense). SHED were cultured in 12-well plates prior to transfection. After reaching 60% confluence, the cells were transfected with siRNAs using Lipofectamine 3000 (Invitrogen, Carlsbad, CA, USA).

### Preparation of dental slices

Caries-free human premolars extracted for orthodontics were obtained. For the in vivo experiments, 2-mm high dentin slices were prepared from the roots of premolars using a hard tissue slicing machine. The root canal was enlarged to prepare the shape of a hollow tube and eliminate the pre-dentin. Before cell seeding and transplantation, the dentin slices were prepared in a sterile tissue culture cabinet. The slices were exposed to ultraviolet light for 30 min, rinsed in phosphate-buffered saline (PBS) three times at 1 min per wash, and immersed in 20 mL 1.25% NaOCl, 17% EDTA, and 0.9% normal saline. After disinfection, the slices were washed in PBS three times.

### Cell seeding and transplantation

The SHED were cultured with α-minimum essential medium supplemented with 10% fetal bovine serum (control group). In another group, IGFBP7-AS1 was upregulated (IGFBP7-AS1 group) as described above. SHED were detached using trypsin–EDTA and resuspended in PBS. The cells (5 × 10^5^) were mixed with 1 mg hydroxyapatite (HA), then incubated at 37 °C with 5% CO_2_ for 3 h.

For the implantation, 6-week-old severe combined immune deficiency (SCID) male mice (CB17) were used as subcutaneous transplant recipients. The groups were as follows: control (dentin slices with normal cultured SHED), and IGFBP7-AS1 (dentin slices with IGFBP7-AS1 overexpression SHED). Six implants were placed per treatment group: two implants per animal on the left and right.

The operations were performed under anesthesia achieved by intraperitoneal injection of 10% pentobarbital sodium. Incisions (approximately 0.5-cm long) were made on each side on the dorsal surface of each animal. Subcutaneous pockets were created by blunt dissection, and one dentin slice was placed in the pocket. The cell–HA compounds were placed in the hollow tube of the dental slice. The incisions were closed with surgical sutures.

The animals were killed 8 weeks after implantation. The implants were fixed in 4% buffered paraformaldehyde solution overnight at 4 °C. After washing in PBS three times, the implants were transferred to 20% EDTA demineralization solution and decalcified for 2 months. The solution was changed daily.

### Histology and immunohistochemistry

For histology, the tissues were stained with hematoxylin and eosin and Masson trichrome to visualize collagen formation (Trichrome Stain [Masson] Kit, Sigma-Aldrich, MO, USA). For immunohistochemistry staining of dentin sialoprotein (DSPP, sc-73632) and dentin matrix acidic phosphoprotein 1 (DMP1, sc-73633), a 1:100 dilution of anti-human antibody was used (Santa Cruz Biotechnology, Santa Cruz, CA, USA).

### RNA isolation and quantitative reverse transcription-PCR (qRT-PCR)

Total RNA was extracted with TRIzol (Invitrogen, Carlsbad, CA, USA), and 1 μg total RNA was converted to complementary DNA using a PrimeScript RT Reagent Kit (TaKaRa, Shiga, Japan). qPCR was performed as previously described [13]. Each genetic analysis was performed in triplicate and the primers used are listed in Table 1.

**Table 1:** The sequences of the qRT-PCR primers used in the study

Gene name		5’ − 3’	Size (bp)	Gene bank number
*GAPDH*	F	CCGTCTTGAGAAACCTGCCA	139	NM_001115114.1
	R	GGATGAACGGCAATCCCCAT		
*ALP*	F	CTCCATACCTGGGATTTCCGC	299	NM_000478.6
	R	GGCCCCAGTTTGTCCTTCTT		
*DSPP*	F	GGAATGGCTCTAAGTGGGCA	284	NM_014208.3
	R	CTCATTGTGACCTGCATCGC		
*DMP1*	F	GAGTGGCTTCATTGGGCATAG	260	NM_004407.4
	R	GACTCACTGCTCTCCAAGGG		
*IGFBP7-AS1*	F	GGTTGGGTTCATGTGCTAC	121	NR_034081.1
	R	AGAATTGCTTCCTGCTAATCT		
*IGFBP7*	F	TGAAGTAACTGGCTGGGTGC	281	NM_001253835.2
	R	CTTAATGCCCTTATGGGTTGC		

### Western blotting

The cells were harvested with protein lysis buffer containing a phosphatase inhibitor (Applygen Technologies Inc., Beijing, China). The cell suspensions were centrifuged at 4 °C for 30 min at 12,000 × *g*. The protein concentration was determined using bicinchoninic acid (BCA) Protein Assay (CWbio, Beijing, China) and each lane was loaded with equal aliquots of total protein (20 μg). The lysates were separated by sodium dodecyl sulfate-polyacrylamide gel electrophoresis, transferred to polyvinylidene difluoride membranes (Millipore, Bedford, MA, USA), blocked in 5% bovine serum albumin for 2 h, and probed with antibodies against the following at 4 °C overnight: IGFBP7 (sc-365293, 1:1000, Santa Cruz Biotechnology, CA, USA), DSPP (sc-73632, 1:1000, Santa Cruz Biotechnology, CA, USA), and β-actin (#4970, 1:10,000, Cell Signaling Technology, Beverly, MA, USA). The membrane was incubated at room temperature for 1 h with horseradish peroxidase-conjugated anti-rabbit immunoglobulin. Protein expression was detected using a western enhanced chemiluminescence blotting kit (Solibro, Beijing, China).

### Alkaline phosphatase (ALP) staining

ALP staining was performed on day 7 of the experiment using an ALP staining kit according to the manufacturer’s protocol (CWbio, Beijing, China). Briefly, the cultured cells were rinsed in PBS and the cell layer was fixed in 4% paraformaldehyde for 30 min. Then, it was washed with distilled water and incubated in an alkaline solution for 10 min at room temperature.

### Alizarin red S (ARS) staining

SHED were rinsed with PBS three times and fixed in 4% paraformaldehyde for 15 min. Then, they were stained with 0.1% ARS (pH 4.0–4.6) for 20 min. Finally, the reaction was terminated with clean water.

### Stability and α-amanitin treatment

SHED and IGFBP7-AS1 overexpression SHED were plated in 6-well plates. The SHED were treated 24 h later with 50 μg/mL α-amanitin and were harvested for PCR at 6, 12, and 24 h post-treatment.

### Statistical analysis

Statistical calculations were performed using SPSS 21.0 (IBM Corp., Armonk, NY, USA). Comparisons between two groups were analyzed using an independent two-tailed Student’s *t* test, and comparisons between > 2 groups were analyzed using one-way analysis of variance followed by Tukey’s post hoc test. All data are expressed as the mean ± SD of three experiments per group, and P < 0.05 was considered statistically significant.

## Results

### IGFBP7-AS1 regulated SHED odontogenesis in vivo

**Fig. 1:**
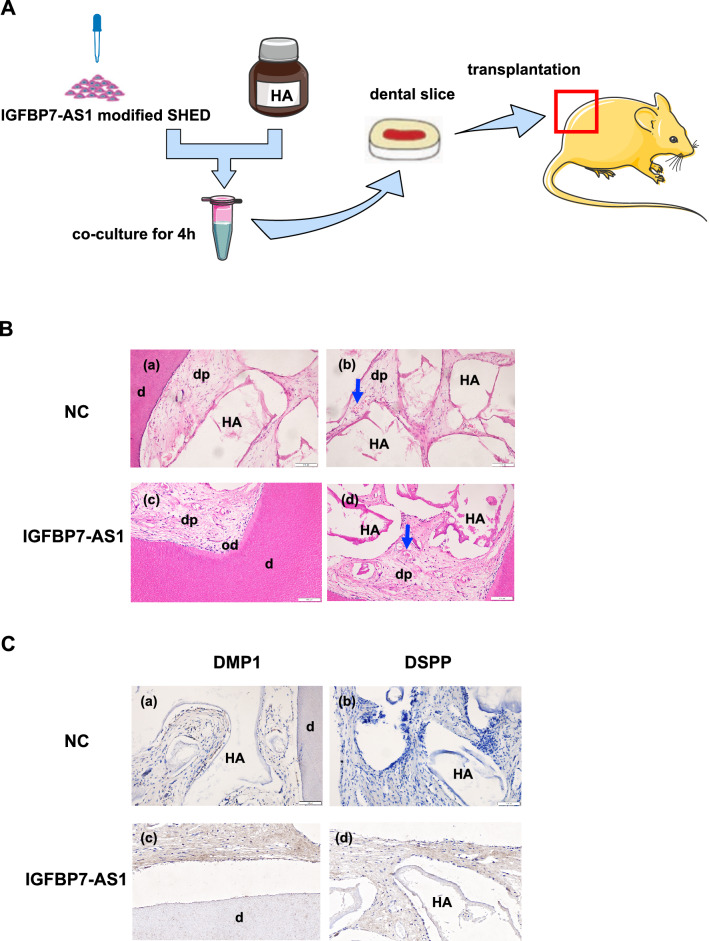
Subcutaneous transplantation results showing that IGFBP7-AS1 promotes SHED odontogenic differentiation in vivo. **A** Dental slice transplantation process. **B** Hematoxylin and eosin staining. NC group (a, b) and IGFBP7-AS1 overexpression group (c, d). Scale bar = 100 μm **C** Immunohistochemistry staining of DMP1(a, c) and DSPP (b, d). Scale bar = 100 μm (*HA* hydroxyapatite, *d* dental slice, *dp* dental pulp, *od* odontoblast; *Represent vessel)

**Fig. 2:**
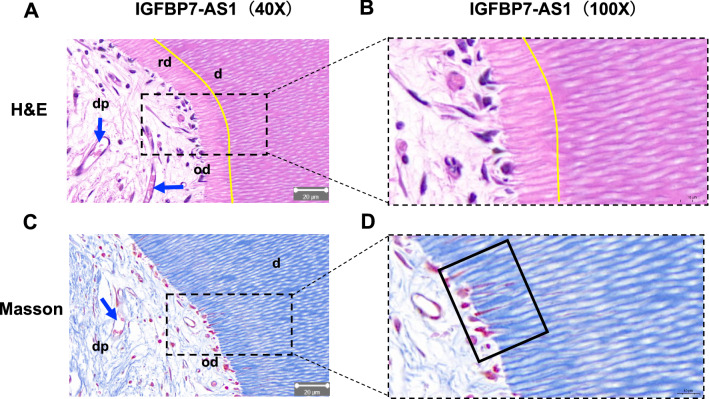
Subcutaneous transplantation results of IGFBP7-AS1 overexpression SHED under high magnification microscopy (40X, Scale bar = 20 μm; 100X, Scale bar = 10 μm). The area stressed by the black line in Masson staining represents the odontoblastic processes. (*d* dental slice, *dp* dental pulp, *rd* regenerated dentin, *od* odontoblast, *vessel)

We conducted animal transplantation experiments to confirm that IGFBP7-AS1 plays a role in regulating SHED odontogenic differentiation. SHED were divided into control and IGFBP7-AS1 overexpression groups. Figure 1A shows the dental slice transplantation process. The cells were loaded on HA scaffolds and implanted in the dorsal surface of SCID mice with the dental slices. Hematoxylin and eosin staining showed the formation of the odontoblast layer in the IGFBP7-AS1 overexpression group (Fig. 1B). Vascularized pulp-like tissues were formed in both the control and IGFBP7-AS1 groups (Fig. 1B). Immunohistochemistry staining of odontogenic-related markers showed that the IGFBP7-AS1 overexpression group had more DSPP and DMP1 formation (Fig. 1C). Moreover, the odontoblast processes extended into the dentinal tubules in the IGFBP7-AS1 overexpression group and this area was emphasized by the black line in Masson staining. The H&E staining also showed the newly formed dentin in the IGFBP7-AS1 overexpression group (Fig. 2).

### IGFBP7-AS1 regulated the expression of *IGFBP7*

**Fig. 3:**
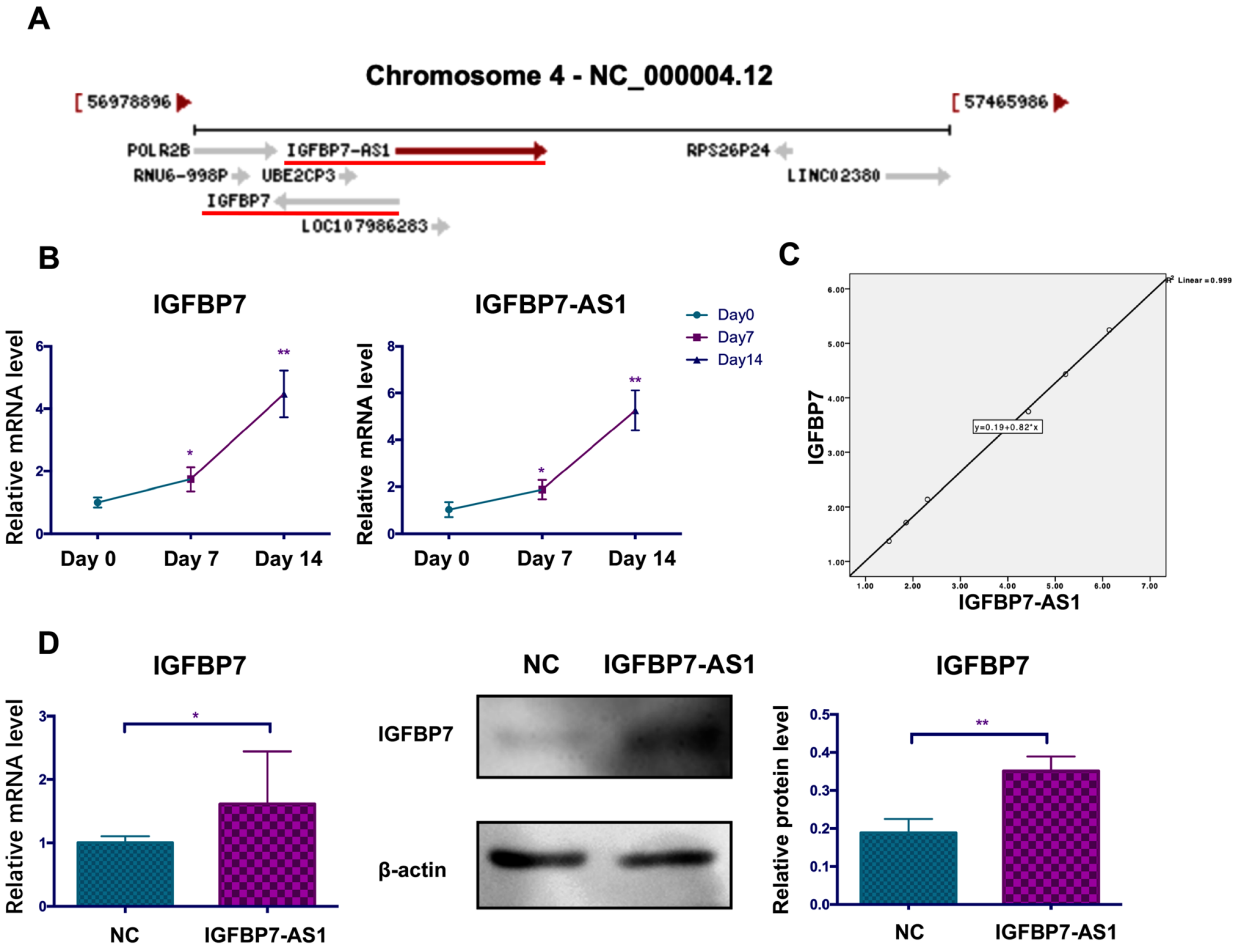
IGFBP7-AS1 regulated the expression of the coding gene *IGFBP7*. **A** The location of IGFBP7-AS1 and *IGFBP7*. **B** qRT-PCR detection of IGFBP7-AS1 and *IGFBP7* expression during odontogenic differentiation on day 0, 7, and 14. **C** Correlation analyses between *IGFBP7-AS1* and *IGFBP7* levels. **D** qRT-PCR and western blot detection of *IGFBP7* expression in the NC and IGFBP7-AS1 overexpression groups. Data were expressed as the mean ± standard deviation (SD) of three experiments per group. *P < 0.05, **P < *0.01*

As IGFBP7-AS1 is located on the strand opposite the coding gene *IGFBP7* (Fig. 3A), which is related to the formation of bone and dental hard tissue, and lncRNAs exert regulatory effects on nearby genes, we investigated whether IGFBP7-AS1 regulates *IGFBP7* expression. Here, *IGFBP7* expression was upregulated during the SHED odontogenic process on day 7 and day 14. Relationship analysis between *IGFBP7-AS1* and *IGFBP7* at the mRNA expression level during odontogenesis yielded a positive correlation (Fig. 3B). Next, we found that IGFBP7 mRNA and protein levels were increased after IGFBP7-AS1 was overexpressed (Fig. 3C). These findings confirm that IGFBP7-AS1 regulates *IGFBP7* expression positively. Additionally, IGFBP7-AS1 expression was unchanged when we knocked down *IGFBP7* (data not shown), which is the evidence that IGFBP7-AS1 is an upstream regulator of *IGFBP7*.

### *IGFBP7* promoted SHED odontogenic differentiation

**Fig. 4:**
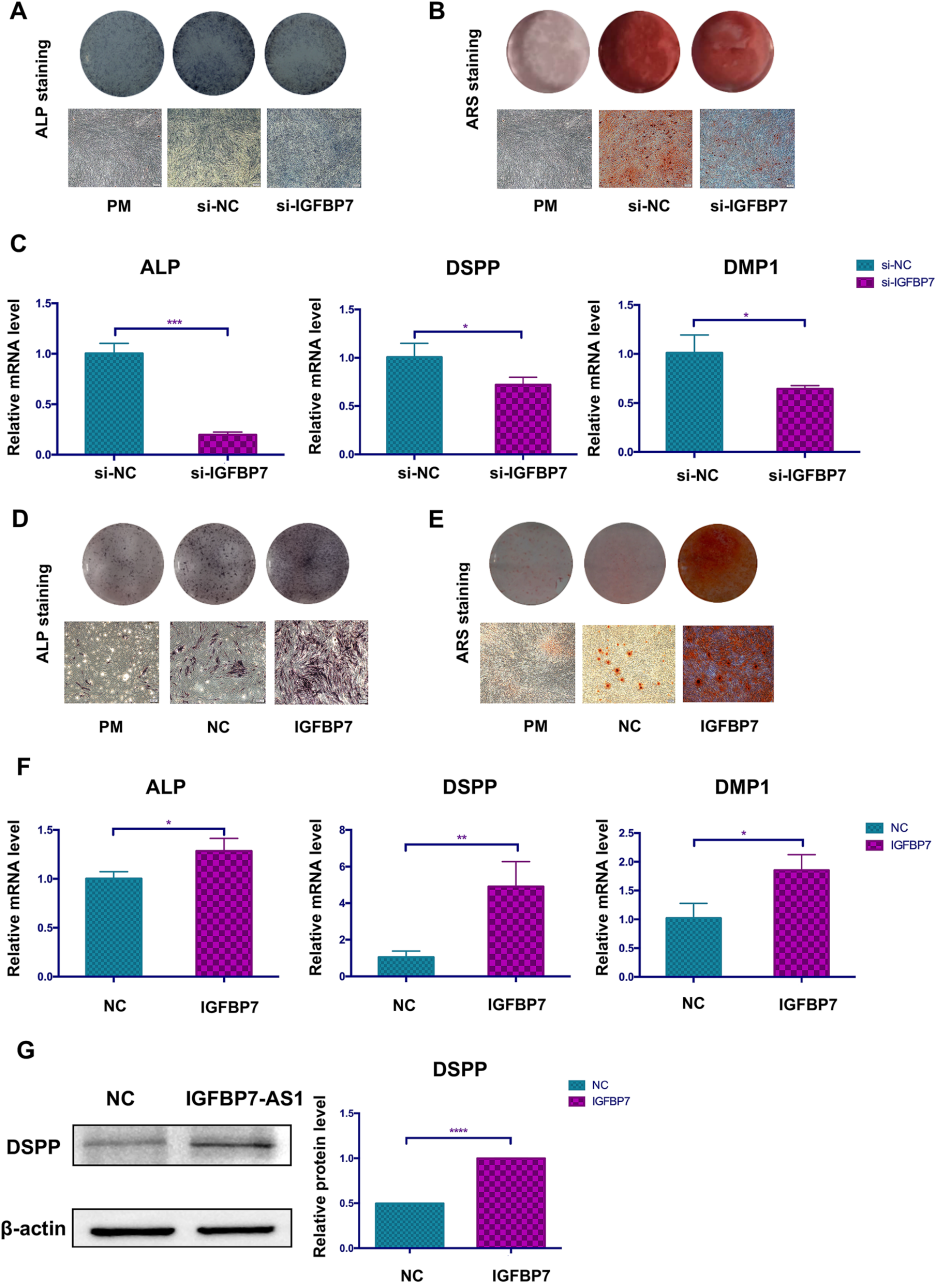
IGFBP7 promoted SHED odontogenic differentiation. **A** Images of ALP staining of SHED on day 7 after odontogenic differentiation in the si-NC and si-IGFBP7 groups. **B** Images of ARS staining of SHED on day 14 after odontogenic differentiation in the si-NC and si-IGFBP7 groups. **C** qRT-PCR detection of the odontogenic differentiation-related markers *ALP, DSPP and DMP1* in the si-NC and si-IGFBP7 groups. **D** Images of ALP staining of SHED on day 7 after odontogenic differentiation in the NC and IGFBP7 groups. **E** Images of ARS staining of SHED on day 14 after odontogenic differentiation in the NC and IGFBP7 groups. **F** qRT-PCR detection of the odontogenic differentiation-related markers *ALP*, *DSPP* and *DMP1* in the NC and IGFBP7 groups. **G** Western blot detection of the odontogenic differentiation-related marker DSPP in the NC and IGFBP7 groups. β-Actin is the internal reference protein. Data were expressed as the mean ± standard deviation (SD) of three experiments per group. *PM* proliferation medium; *P < 0.05, **P < 0.01, ***P < 0.001, ****P < *0.0001*

To determine the effect of IGFBP7 on SHED odontogenic differentiation, lentiviruses were used to overexpress *IGFBP7-AS1*, and *IGFBP7* expression was knocked down with siRNA. Successfully transfected SHED were cultured in OM and harvested on days 7 and 14. The ALP staining showed that ALP expression levels were decreased by *IGFBP7* knockdown and increased by *IGFBP7* overexpression (Fig. 4A, D). The same tendency was observed in ARS staining (Fig. 4B, E). ALP and DSPP are odontogenic markers and their expression was assessed in SHED cultured in OM for 14 days. *IGFBP7* knockdown significantly downregulated *ALP* and *DSPP* mRNA levels (Fig. 4C). However, *IGFBP7* overexpression markedly upregulated the expression of these genes (Fig. 4F). We confirmed that IGFBP7 regulates the odontogenic differentiation-related marker DSPP at protein level (Fig. 4G).

### IGFBP7-AS1 enhanced the stability of *IGFBP7*

**Fig. 5:**
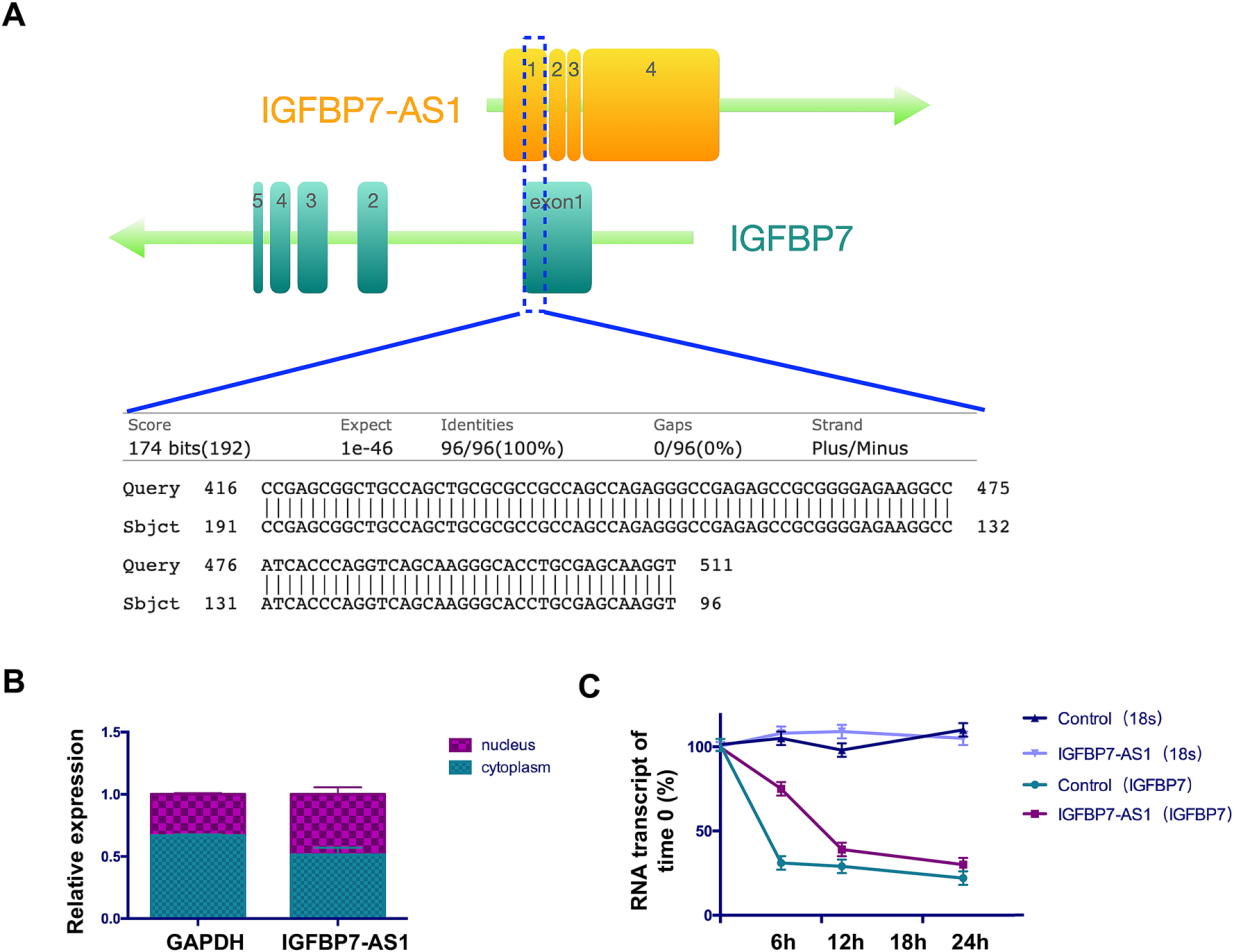
IGFBP7-AS1 enhances the stability of *IGFBP7*. **A** IGFBP7-AS1 has 96-bp nucleotides complementary to the *IGFBP7* exon 1 region. **B** PCR detection of the subcellular fractionation of *IGFBP7-AS1*. **C** PCR detection of *18S* RNA and *IGFBP7* expression after α-amanitin treatment. Data were expressed as the mean ± standard deviation (SD) of three experiments per group

The National Center for Biotechnology Information database showed that the IGFBP7-AS1 sequence mapped to *IGFBP7*, which is on the opposite strand of IGFBP7-AS1. IGFBP7-AS1 has 96 bp nucleotides complementary to the *IGFBP7* exon 1 region (Fig. 5A). To explore the underlying mechanism of IGFBP7-AS1 regulation of *IGFBP7*, we conducted subcellular fractionation of IGFBP7-AS1 via qRT-PCR. The result showed that the presence of IGFBP7-AS1 in the cytoplasm was greater than that in the nucleus at a ratio of 60%:40% (Fig. 5B). Antisense lncRNAs regulate the mRNA stability of the gene on the opposite strand through a sense:antisense hybrid. We used ViennaRNA (http://rna.tbi.univie.ac.at) to predict the minimum free energy (MFE) of RNA hybridization [14]. The results showed that the MFE of *IGFBP7*-*AS1*:*IGFBP7* duplexes was − 1448.40 kcal/mol, much less than that of *IGFBP7* or *IGFBP7-AS1* individually (*IGFBP7*: − 449.40 kcal/mol; *IGFBP7*-*AS1*: − 851.20 kcal/mol). These results indicate that IGFBP7-AS1 may enhance the stability of *IGFBP7*. To confirm the bioinformatics prediction, we assessed IGFBP7-AS1 and *IGFBP7* stability by blocking new RNA synthesis using α-amanitin and measuring their expression levels during a 24-h period. 18S RNA, a product of RNA polymerase I, was not affected during the period and was used as a control. IGFBP7-AS1 and *IGFBP7* expression was downregulated after the α-amanitin treatment. SHED overexpressing IGFBP7-AS1 had increased *IGFBP7* mRNA stability compared with other groups (Fig. 5C). The results confirm the prediction and IGFBP7-AS1 enhances the stability of *IGFBP7*.

## Discussion

SHED have the potential for multidirectional differentiation [15]. Hence, they are considered good seed cells for tissue engineering due to their ability to develop into neural cells, adipocytes, and osteoblasts [1]. Moreover, they express odontogenesis-related markers such as DSPP and DMP1, and differentiate into odontoblast-like cells in vivo [16]. The use of SHED in odontogenic differentiation may be advantageous over traditional mesenchymal stem cells for the following reasons: (1) SHED have a higher rate of proliferation in vitro than human bone marrow mesenchymal stem cells (hBMSCs) [17] or dental pulp stem cells (DPSCs) [18]; (2) SHED have a greater capacity to form dentine than other bone marrow derived mesenchymal stem cells like hBMSCs. [1]; and (3) SHED are isolated from the disposable organ: deciduous teeth, which is associated with fewer ethical concerns. Emerging evidence indicates that lncRNAs may play crucial roles in regulating differentiation and stem cell biology, and may be key regulators in human hard tissue regeneration [19, 20]. It has been confirmed that some lncRNAs, such as DANCR and H19, regulate odontogenic differentiation through different pathways. One study explored lncRNA differential expression during the osteogenic/odontogenic differentiation of dental pulp stem cells (DPSCs) and found that the lncRNA SNHG7 may be a target in osteo/odontoblast differentiation of DPSCs [21]. Previously, our RNA sequencing results showed that *IGFBP7*-*AS1* was upregulated during SHED odontogenic differentiation. This indicates that IGFBP7-AS1 could be a good target for SHED in the regeneration of dental hard tissue.

Pulp–dentin complex formation is crucial for regenerating dental hard tissue. The deposition of new dentin on the existing dentin surface and the formation of a layer of odontoblast-like cells are considered necessary for regenerated pulp–dentin complex identification [22]. IGFBP7-AS1 may be a target for regulating dental hard tissue regeneration at the gene level. In the present study, we performed subcutaneous transplantation experiments in nude mice to verify the effect of IGFBP7-AS1 in vivo. It is important for seed cells to differentiate into odontoblasts to generate new dentin. The results showed that SHED in the IGFBP7-AS1 overexpression group were able to differentiate into odontoblast-like cells lining against the existing dentin surface and form dentin-like tissue. We also observed the cytoplasmic processes extended into the dentinal tubules in IGFBP7-AS1 overexpression group, which indicates the formation of odontoblast-like cells. Immunohistochemistry also showed higher expression of the odontogenic-related marker DSPP in the IGFBP7-AS1 overexpression group. Furthermore, both vascularized pulp-like tissues were formed in the IGFBP7-AS1 overexpression group and control group [23]. This indicates that SHED have the ability to regenerate pulp tissue on their own, which is consistent with previous study. Nevertheless, IGFBP7-AS1 presented the capacity for promoting SHED odontogenic differentiation in vivo.

IGFBP7-AS1 belongs to natural antisense RNAs and has been significantly associated with overall survival in patients with glioblastoma [24]. Natural antisense RNAs constitute a group of lncRNAs that are transcribed from the opposite strand as compared to sense transcripts, with partial or complete complementarity. They influence the expression of their sense genes, such as influencing transcription or regulating post-transcriptional processing. From recent studies, it is worth noting that antisense lncRNAs can promote the expression of their sense gene by stabilizing the mRNA [25–27]. BACE1-AS is an antisense lncRNA and is elevated in Alzheimer disease. It increases the stability of its sense gene mRNA, i.e., β-secretase 1 (*BACE1*), by forming duplex RNA and generating more amyloid-β through a post-transcriptional positive feedback mechanism [28]. Another study reported that the lncRNA BAZ2B promotes M2 macrophage activation and inflammation by forming a duplex with pre-mRNA *BAZ2B*, increasing the stability of pre-mRNA *BAZ2B* and further stabilizing the mature mRNA [29]. This suggests that IGFBP7-AS1 may also regulate SHED odontogenic differentiation by regulating its sense gene *IGFBP7.* Interestingly, we found that IGFBP7-AS1 was expressed concordantly with *IGFBP7* during SHED odontogenic differentiation. Furthermore, IGFBP7-AS1 is the upstream regulator of *IGFBP7* mRNA. IGFBP7 has been associated with regions of bone and dentine deposition [12]. It is also related to extracellular matrix and cell adhesion, which are important in osteo/odontogenic differentiation [30, 31]. Here, we confirmed that *IGFBP7* improved SHED odontogenic differentiation in vitro. Exploration of the mechanism of IGFBP7-AS1 regulation of *IGFBP7* expression showed that IGFBP7-AS1 and *IGFBP7* were partially complementary in the exon 1 region with 96 bp. IGFBP7-AS1 may improve the stability of *IGFBP7*. Bioinformatics analysis calculating the RNA hybridization MFE [14] showed that the IGFBP7-AS1 and *IGFBP7* combination is more stable than that of a single transcript. To verify these results, we added α-amanitin, which blocks new RNA synthesis, to SHED overexpressing IGFBP7-AS1 or *IGFBP7*. IGFBP7-AS1 improved the stability of *IGFBP7* mRNA after α-amanitin treatment. Therefore, we conclude that IGFBP7-AS1 regulates SHED odontogenic differentiation by upregulating the stability of *IGFBP7*.

In this study, we verified the effect of IGFBP7-AS1 in SHED odontogenic differentiation in vivo and explored the mechanism of IGFBP7-AS1 regulation thereof by improving the stability of *IGFBP7*. This could provide a therapeutic target in dental regeneration. What’s more, we used lentivirus to up-regulate the expression of IGFBP7-AS1 in SHED; however, lentivirus-based cells cannot be used in regenerative medicine. In the future, we should explore the feasible way to up-regulate the expression of IGFBP7-AS1in SHED to immortalize seed cells.
